# Comparative Analysis of QCM and Electrochemical Aptasensors for SARS-CoV-2 Detection

**DOI:** 10.3390/bios14090431

**Published:** 2024-09-06

**Authors:** Katarína Nemčeková, Jana Korčeková, Veronika Svitková, Denis Baraniak, Michaela Domšicová, Eva Melníková, Michaela Hornychová, Viktória Szebellaiová, Miroslav Gál, Alexandra Poturnayová

**Affiliations:** 1 Department of Inorganic Technology, Faculty of Chemical and Food Technology, Slovak University of Technology in Bratislava, 812 37 Bratislava, Slovakia; katarina.nemcekova@stuba.sk (K.N.); veronika.svitkova@stuba.sk (V.S.); eva.melnikova@stuba.sk (E.M.); viktoria.szebellaiova@stuba.sk (V.S.); 2 Institute of Analytical Chemistry, Faculty of Chemical and Food Technology, Slovak University of Technology in Bratislava, 812 37 Bratislava, Slovakia; denis.baraniak@stuba.sk; 3 Center of Biosciences, Institute of Molecular Physiology and Genetics, Slovak Academy of Sciences, 840 05 Bratislava, Slovakia; jana.korcekova@savba.sk (J.K.); michaela.domsicova@savba.sk (M.D.)

**Keywords:** aptasensor, QCM, SARS-CoV-2, electrochemical impedance spectroscopy

## Abstract

The rapid and accurate detection of SARS-CoV-2, particularly its spike receptor-binding domain (S-RBD), was crucial for managing the COVID-19 pandemic. This study presents the development and optimization of two types of aptasensors: quartz crystal microbalance (QCM) and electrochemical sensors, both employing thiol-modified DNA aptamers for S-RBD detection. The QCM aptasensor demonstrated exceptional sensitivity, achieved by optimizing aptamer concentration, buffer composition, and pre-treatment conditions, with a limit of detection (LOD) of 0.07 pg/mL and a linear range from 1 pg/mL to 0.1 µg/mL, and a significant frequency change was observed upon target binding. The electrochemical aptasensor, designed for rapid and efficient preparation, utilized a one-step modification process that reduced the preparation time to 2 h while maintaining high sensitivity and specificity. Electrochemical impedance spectroscopy (EIS) enabled the detection of S-RBD concentrations as low as 132 ng/mL. Both sensors exhibited high specificity, with negligible non-specific interactions observed in the presence of competing proteins. Additionally, the QCM aptasensor’s functionality and stability were verified in biological fluids, indicating its potential for real-world applications. This study highlights the comparative advantages of QCM and electrochemical aptasensors in terms of preparation time, sensitivity, and specificity, offering valuable insights for the development of rapid, sensitive, and specific diagnostic tools for the detection of SARS-CoV-2 and other viruses.

## 1. Introduction

The global pandemic caused by severe acute respiratory syndrome coronavirus 2 (SARS-CoV-2) has necessitated the rapid development and deployment of reliable diagnostic tools. The early and accurate detection of the virus was of critical importance for the effective management and containment of the disease. Among the various diagnostic approaches, biosensors have emerged as a promising technology due to their high sensitivity and specificity and rapid response times [1,2].

Quartz crystal microbalance (QCM) is a powerful, non-invasive, surface-sensitive, and label-free technique allowing the monitoring of interactions between adsorbed lipid layers and proteins or adhesive interactions between cells and the sensor surface directly in real time. This method is often used to study antibody and aptamer interactions with target molecules or whole cells [3,4,5]. QCM sensing is based on a piezoelectric quartz crystal (QMC) sensor that oscillates at the defined frequency when an appropriate voltage is applied via metal electrodes. The crystals used in QCM are typically AT-cut, ensuring high stability at room temperature and minimal frequency changes. The frequency of oscillation is changed upon binding or dissociation of small mass on or from the crystal surface. This change in frequency is monitored over time to obtain information about molecular interactions or reactions at the crystal surface. In the proposed experiments, chemically sensitive layers will be immobilized on the surface of a gold electrode of the QCM sensor. The addition of proteins or real samples in the flow regime will cause current-measured changes in the frequency of piezocrystal oscillations. We expect that this interaction will lead to a decline in frequency change as a consequence of an increase in the weight of a sensitive layer [6,7,8].

Aptamers are short, single-stranded DNA or RNA molecules that exhibit high affinity and specificity in binding to specific targets. The advantages include the ease of synthesis, modification, and stability, which are superior to those of traditional antibodies. In the context of SARS-CoV-2 detection, aptamers have been employed to develop a variety of biosensing platforms, with QCM and electrochemical sensors being among the most extensively studied [7,9].

Due to their three-dimensional structure, acquired in the liquid phase, aptamers form a specific binding site for the particular molecule [10]. The binding properties of aptamers are contingent upon their three-dimensional structure rather than their primary sequence. The manner of production enables them to bind a variety of molecules, including simple organic compounds, large protein complexes, and whole cells [11]. Aptamers are gaining increasing popularity, with recent studies on aptamers sensitive to pathogens, including virus particles of immune deficiency HIV-1, hepatitis type C, and influenza, emerging in the literature [12,13,14,15,16]. The utilization of aptamers in biosensors (aptasensors) is becoming increasingly diverse across various fields and sectors. At present, aptasensors that are capable of recognizing ochratoxin A (technology OTA-Sense, NeoVentures Biotechnology, London, ON, Canada) or aflatoxin (technology AflaSense, NeoVentures Biotechnology) are available for commercial use. They serve as detection components in a diagnostic kit for several biomarkers (AptoPrep, AptSci Inc., Seongnam-si, Republic of Korea). Aptamer, AS1411, which is utilized for the identification of nucleolin on the surface of cancer cells, has reached the second phase of clinical trials [17]. The potential for the translation of aptasensors into clinical practice is considerable. The appealing characteristics of these devices encourage researchers to persist in the refinement of their implementation within the biomedical domain. This could facilitate the further commercialization of aptamers and biosensors in clinical practice in the near future [18].

The QCM aptasensor operates on the principle of mass sensitivity, whereby the binding of the target virus to the aptamer results in a quantifiable alteration in the resonance frequency of a quartz crystal. This method allows for label-free detection and provides the real-time monitoring of binding events. In contrast, electrochemical aptasensors detect the presence of the virus through changes in electrical signals, such as current or potential, generated by the binding interactions at the sensor surface [19,20,21,22]. These sensors are known for their high sensitivity, rapid response, and potential for miniaturization and integration into portable devices [18,23,24].

In general, the widely available and reliable testing of a broad population is the key step for better understanding the prevalence, incidence of asymptomatic infection, and identification of potential spreaders of a virus in the fight against a pandemic and its sociological, economic and cultural consequences in the current situation with no effective pharmacological treatment or vaccines [25]. The sensitive detection of virus markers using new diagnostic approaches could, therefore, fill the diagnostic gap and reliably reveal asymptomatic spreaders of infection. Despite the extensive research and development in both QCM and electrochemical aptasensors, a direct comparison of their performance in the detection of severe acute respiratory syndrome coronavirus 2 (SARS-CoV-2) is lacking. This study aims to address this gap by evaluating the sensitivity, specificity, response time, and practical applicability of both sensor types under similar experimental conditions. The primary aim of this work is to develop a sensitive diagnostic method based on DNA aptamer interactions with structural proteins characteristic of the SARS-CoV-2 virus located in the lipid membrane of virus particles and ensure interaction with receptors in the plasma membrane of host cells.

## 2. Materials and Methods

Neutravidin (NA) and Tris(2-carboxyethyl)phosphine hydrochloride (TCEP) were purchased from Thermo Fischer Scientific GmbH (Dreieich, Germany), and 1-dodecanethiol, recombinant spike RBD protein (S-RBD), nucleocapsid proteins (N-protein), and 6-mercapto-1-hexanol (MCH) were purchased from Sigma-Aldrich (Bratislava, Slovakia). Inorganic salts, ethanol, methanol, acetone, hydrogen peroxide, ammonia, and sulfuric acid (p.a.) were obtained from CentralChem (Bratislava, Slovakia). Ultrapure water with a resistivity of 18.2 MΩ cm at 25 °C was used (Simplicity 185, Merck Millipore, Burlington, MA, USA).

**Figure 1:**
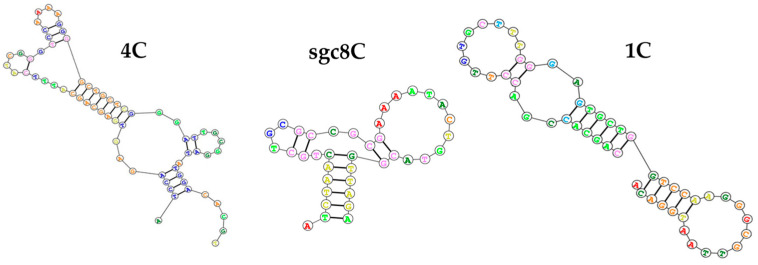
Sequences of DNA aptamers used in this study. The sequences were generated by https://rna.urmc.rochester.edu/ (accessed on 20 July 2024).

The DNA aptamers (1C, 4C, and sgc8c) modified at the 5′-end by thiol were purchased from MicroSynth (Balgach, Switzerland). The aptamer sequences (Figure 1) were taken from Refs. [26,27]. Aptamers 1C and 4C were selected based on already available and known transmembrane protein S-RBD recognition sequences tested by Song et al. [26] in their study. The sgc8c aptamer was available in our laboratory as a non-specific sequence of similar length that recognizes PTK7 (protein tyrosine kinase-7) transmembrane receptors expressed in multiple tumor cell types [27]. The aptamer stock solutions were prepared by dissolving lyophilized oligonucleotides in TE buffer (1 mM EDTA, 10 mM Tris, pH 8). After dilution of the aptamers in binding buffer to the desired concentration, the aptamer was heated for 3 min at 95 °C, cooled for 10 min on ice, and then slowly warmed to laboratory temperature. The aptamer solutions prepared following the aforementioned methodology were subsequently utilized for the measurements.

### 2.1. QCM Setup and Preparation of Aptasensors

As a binding buffer, phosphate-buffered saline (PBS) with 0.55 mM MgCl_2_ (10 mM phosphate-buffered saline: 10 mM NaH_2_PO_4_, 1.8 mM KH_2_PO_4_, 137 mM NaCl, and 2.7 mM KCl, pH 7.4) was used.

The detection of S-RBD protein in human plasma was performed in the same concentration range as in PBS. Lyophilized human plasma norm-Trol 1 samples were acquired from Helena BioSciences (Gateshead, UK) and diluted 10 times by PBS + 0.55 mM MgCl_2_. The saliva was diluted 10-fold in PBS and, like the plasma, filtered through a 0.22 μm syringe membrane filter. The samples were then enriched with a known concentration of SARS-CoV-2 and added to the surface of the aptasensor.

For the preparation of the aptasensor, we used AT-cut quartz with a fundamental frequency of 10 MHz (QuartzPro, Jarfalla, Sweden), covered on both sides by polished gold electrodes. The frequency was measured with 0.1 Hz accuracy. The syringe pump Genius Plus (Kent Scientific, Torrington, CT, USA) was used for the addition of washing buffers and samples onto the crystal surface with a constant flow rate of 50 μL/min. The oscillation frequency changes were measured by Maxtek RQCM (Inficon, Bad Ragaz, Switzerland).

Before the deposition of the sensing layer on one side of the quartz crystal, it was necessary to ensure that the gold layer was accurately cleaned. To achieve this, chemical cleaning using a basic Piranha solution was employed. The crystal was immersed in a hot mixture of basic Piranha, composed of ammonium, water, and hydrogen peroxide in a volume ratio of 1:5:1 at 70 °C for 25 min, followed by rinsing in MilliQ water. This process was repeated three times. Subsequently, the crystal was washed with a copious volume of distilled water, and then with 90% ethanol, and finally dried in a stream of nitrogen gas. The purified crystal was then mounted into the flow cell, thus rendering it ready for further modification [24].

**Figure 2:**
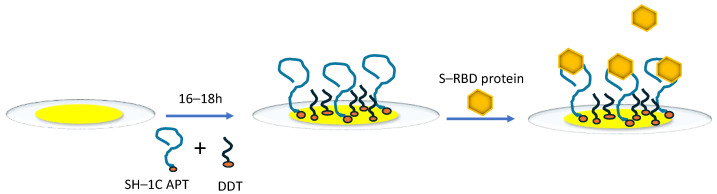
Schematic illustration of QCM aptasensor preparation.

QCM aptasensors were employed as follows: The mixture of 0.5 μM thiolated 1C aptamers and 1 mM dodecanethiol (DDT) was added to the surface of a clean quartz crystal and incubated 16–18 h overnight in an incubation cell. DDT was employed as a filling agent to prevent non-specific interactions between the aptamers and gold surface, which could otherwise occur due to the presence of vacancies between the aptamers. After rinsing the sensing surface with a PBS buffer containing 0.55 mM MgCl_2_, using firstly a pipette and then the constant flow, it was ready for experiments with proteins (Figure 2).

All experiments were performed at an ambient temperature of 23 °C. It should be noted that the concentration of aptamers (0.5 μM) was selected based on optimization experiments in which the response of the sensors based on aptamers was analyzed for various concentrations of aptamers (0.25; 0.5; 1 μM). The results demonstrated that the surface formed by 0.5 μM aptamers exhibited the most notable changes in the resonant frequency (see Figure S1), which was in correspondence with our previous results [23,28], and therefore was used for other experiments.

### 2.2. Electrochemical Setup and Aptasensor Preparation

The electrochemical approach was used for fast, sensitive, and selective S-RBD detection as it has been demonstrated to be useful for the detection of host–guest (aptamer/DNA-virus/protein) interactions at the electrode surface [19,20,21]. In this work, a three-electrode system consisting of a gold working electrode (AuE), reference argentochloride electrode (Ag/AgCl), and auxiliary platinum electrode was used for the electrochemical setup. Signals were measured using an Autolab PGSTAT-100 instrument from Metrohm Autolab (Utrecht, The Netherlands), and the measured data were obtained using the NOVA program 1.11.

A clean and high-quality electrode surface is crucial for reliable aptasensor performance. We followed the cleaning protocol published by Plaxco et al. [29]. In the first step of cleaning in a solution of 0.5 mol/L NaOH, the electrode was exposed to a high scanning speed of 2 V/s, (−0.35 V; −1.35 V) for 1000 scans. After that, chronoamperometry in 0.5 mol/L H_2_SO_4_ in 2 steps was performed: at +2 V for 5 s and −0.35 V for 10 s. Cyclic voltammetry (CV) was again performed under the following conditions: (−0.35 V; +1.5 V); 4 V/s; 20 scans and (−0.35 V; +1.5 V); 0.1 V/s; 4 consecutive CV scans. The electrode was considered clean when the shape of the CV curve stabilized during cleaning, and no increase or decrease of redox peaks was observed.

Electrode modification was performed based on the protocol by Xiao et al. [29], with some optimizations. The modification solution consisting of thiol-modified aptamer and 10 mM TCEP was incubated for 1 h. After the activation of the thiol functional group, the electrode was immersed in this solution for 2 h. During this time, the aptamer molecules were covalently bound to the gold surface. A 2 mM MCH solution was then applied to block the vacancies on the surface, and the electrode was incubated overnight. The entire process was performed in the dark at room temperature, taking approximately 12 h.

**Figure 3:**
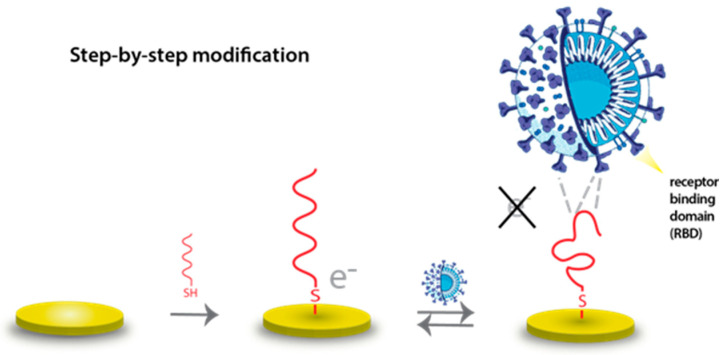
Schematic illustration of one-step electrochemical aptasensor preparation.

To reduce the extensive time commitment required for electrode modification, we optimized the procedure based on literature findings [30]. The time needed to minimize non-specifically adsorbed aptamers and block active sites on the electrode with MCH was reduced by mixing all of the modifying solutions (thiol-modified aptamer, 10 mM TCEP, 2 mM MCH, and PBS—the same as for QCM) and incubating for only 2 h. This optimization significantly reduced the preparation time for the aptasensor without compromising its performance (Figure 3).

All of the data were verified on multiple devices across our laboratories.

## 3. Results and Discussion

### 3.1. Optimization of QCM Aptasensor Preparation

To optimize the sensor surface modification, various buffer types were tested. Among these, the aptamers prepared in PBS with 0.55 mM MgCl_2_ at pH 7.4 exhibited the best sensitivity and stability. Therefore, this buffer was selected for further experiments [31].

The next phase focused on determining the optimal concentration, DNA sequence, and the impact of temperature on the aptamer layer preparation. Aptamer concentrations of 0.25, 0.5, and 1 µM were tested. After the incubation of individual aptamers with DDT overnight, the surfaces were washed with PBS buffer, and the sensitivity and specificity were evaluated using S-RBD protein and N-protein at concentrations ranging from 1 pg/mL to 1 µg/mL. The highest sensitivity was observed at a thiol-1C aptamer concentration of 0.5 µM (see Figure S1).

**Figure 4:**
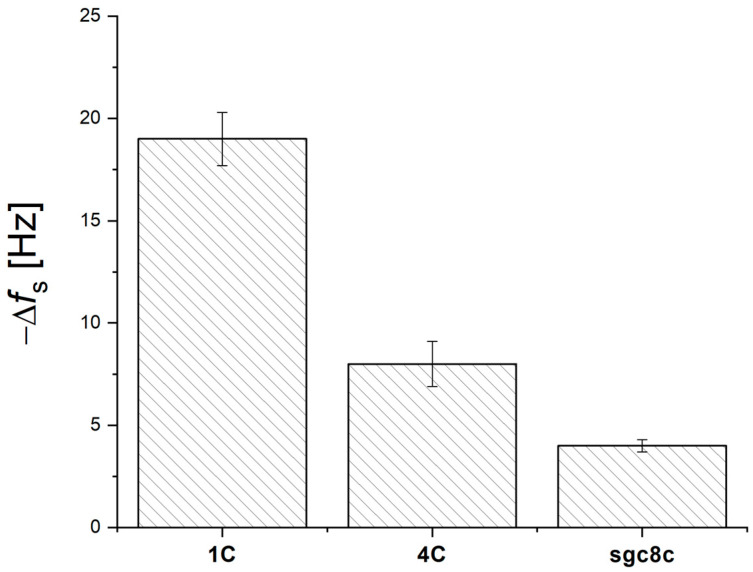
Comparison of changes in resonance frequency of aptasensors prepared from individual aptamer sequences after the addition of 10 pg/mL S-RBD protein.

Figure 4 compares changes in resonant frequency for different aptamer sequences. The 1C sequence showed the largest response, while the 4C aptamer showed lower or no frequency changes. The specificity of the proposed DNA aptamer-based biosensors was evaluated using a non-specific aptamer (sgc8c) for recognizing S-RBD protein. No significant frequency changes were observed, indicating that the modifications were specific (Figure 4). To further exclude non-specific interactions, S-RBD protein was also tested in the presence of neutravidin and dodecanethiol without immobilized aptamers. High concentrations of aptamers could cause steric hindrance, preventing effective binding, whereas lower concentrations resulted in insignificant sensor responses due to fewer binding sites. At a concentration of 10 ng/mL, the interaction resulted in a decrease of 1.5 ± 0.6 Hz for DDT, which was nearly indistinguishable from instrument noise Non-specific interactions represented 1.3% of the total specific response of the HS-1C + DDT aptasensor.

Increasing the temperature can induce thermal denaturation of the DNA, and slowly cooling the solution may facilitate the recovery of the secondary structure and a potential change in the conformation of the aptamer and, consequently, improve or decrease aptasensor performance. In several published papers, the authors describe the higher efficiency of an aptamer that has undergone thermal shock. However, the effect of this phenomenon is usually dependent on the primary structure and the complexity of the three-dimensional conformation [32,33,34,35]. As part of the biosensor optimization process, experiments with heated (95 °C, and then cooled on ice and gradually warmed to room temperature) and unheated aptamers were conducted. In our case, heated 1C aptamers showed a 39 Hz higher change in *f_s_* after adding 1000 pg/mL of S-RBD protein compared to unheated aptamers (see Figure S2).

**Figure 5:**
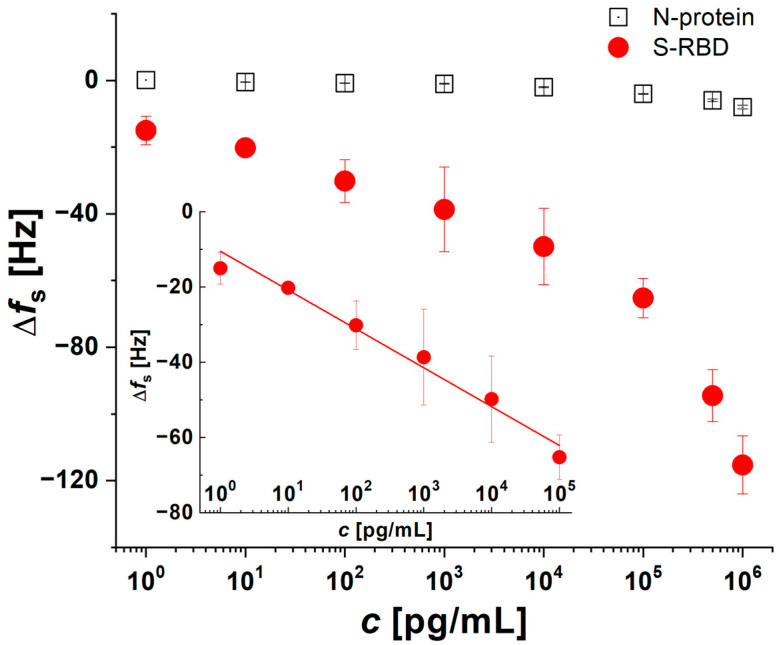
Dependence of Δ*f_s_* on S-RBD (red circle) and N-protein (hollow black square) concentration for sensors based on the thiol-1C aptamer. Mean values of ± SD for 3 measurements in each run are shown.

The responses of the most sensitive 1C aptamers to S-RBD were summarized in calibration curves, represented by the changes in *f_s_* as a function of S-RBD and N-protein concentration. Signal changes were recorded in the concentration range of 1 pg/mL to 1 µg/mL and the linear range was from 1 pg/mL to 0.1 μg/mL (Figure 5).

An aptamer layer was formed by incubating HS-aptamers in a mixture with 1 mM dodecanethiol. As illustrated in Figure 5, the change in frequency shifts downward with an increase in protein concentration. The plot for S-RBD protein exhibits the characteristics of typical isotherms, demonstrating saturation at higher protein concentrations. In contrast to the observations made with S-RBD, the data indicate that there are minimal changes in Δ*f_s_* when analyzing N-protein. As illustrated in Figure 5, the sensing layer, comprising thiolated 1C aptamers, displays a high degree of sensitivity to low concentrations. At a concentration of 1 pg/mL of S-RBD, notable changes in Δ*f_s_* were observed, with a value of 15.0 ± 4.2 Hz. The addition of a maximal concentration of 1 µg/mL of S-RBD resulted in a higher sensor response, Δ*f_s_* = 115.0 ± 8.7 Hz, in comparison with N-protein at the same concentration. From the calibration curve (Figure 5 inset; $\Delta f_{s}=-10.491\pm 1.318-\left(10.331\pm 0.832\right)\;c;\;R^{2}=0.975$; linear range from 1 pg/mL to 0.1 μg/mL), the limit of detection (LOD) was determined to be 0.07 pg/mL, while the limit of quantification (LOQ) was found to be 0.25 pg/mL.

### 3.2. Verification of the Functionality and Stability of QCM Aptasensors in Biological Fluids

**Figure 6:**
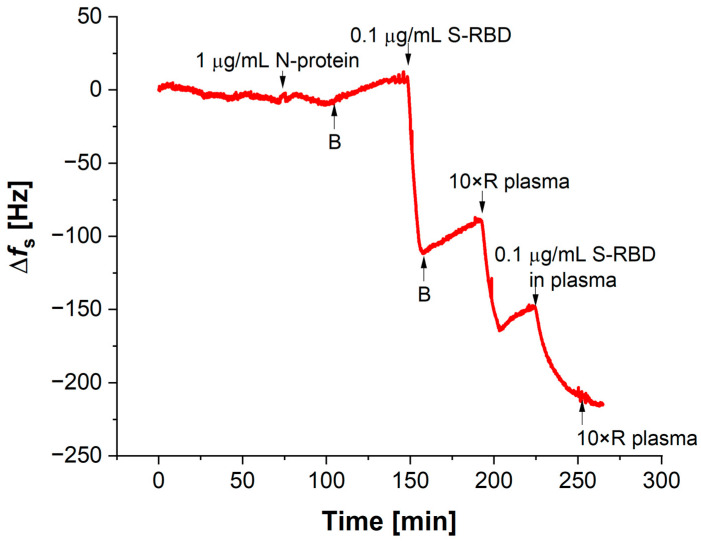
The changes in Δ*f_s_* in the dependence of non-specific interaction with N–protein (1 µg/mL) and, subsequently, the specific response to the addition of 0.1 µg/mL of S—RBD protein. B denotes washing with buffer. Individual additions are marked with arrows.

The functionality of the aptasensors was verified in diluted human plasma and saliva to ensure performance in complex biological environments. To detect S-RBD in real samples, sensors were washed with diluted plasma or saliva to avoid interference from sample components. After stabilization, a solution of diluted plasma enriched with S-RBD protein was added to the sensor. The mixture of 0.5 µM thiol-1C and 1 mM DDT was used for its optimal sensitivity and stability. Figure 6 shows the non-specific interaction with 1 µg/mL N-protein and the specific response to 0.1 µg/mL S-RBD protein, demonstrating the sensor’s functionality and stability.

The initial step involved the incorporation of N-protein into the aptasensor, with the objective of evaluating the sensor’s nonspecific response. As illustrated in Figure 5, there was no observable alteration in the frequency signal. This indicates that there is no interaction between the N-protein and the aptasensor. Subsequently, the sensor was washed with buffer (B in Figure 6) and S-RBD protein was added. Following the addition of S-RBD, a notable decrease in frequency was observed, indicative of the binding of S-RBD to the aptasensor. The magnitude of the signal change was found to be proportional to the concentration of the added S-RBD (see Figure 5). After the interaction with S-RBD, the aptasensor was subjected to washing with diluted plasma, after which the sensor’s capacity to re-detect S-RBD protein in plasma was re-verified.

To ascertain the feasibility of employing this aptasensor for the detection of S-RBD in authentic samples, we conducted an analysis of the aptasensor’s response in plasma and saliva. Control experiments conducted in PBS were performed to ascertain the aptasensor’s recovery by comparison of the QCM response. Table 1 illustrates the S-RBD concentration in spiked 10× diluted plasma or saliva, as determined by changes in resonant frequency.

The response of the aptasensor following the addition of the S-RBD in plasma and saliva samples, as well as those in PBS, was compared. The sensor recovery (*µ*) was determined by Equation (1):

(1) $$\mu =\frac{{\left(\Delta f_{s}\right)}_{PBS}}{{\left(\Delta f_{s}\right)}_{plasma/saliva}}\times 100\%$$

where (Δ*f_s_*)*_PBS_* represents changes in resonant frequency following the addition of S-RBD at a specified concentration in PBS, and (Δ*f_s_*)*_plasma/saliva_* represents those in plasma or saliva. At lower concentrations of S-RBD, the aptasensor demonstrated a recovery of 97.4 ± 5.1% and 93.6 ± 3.6%, respectively. For the higher concentration of 10^5^, the matrix effect increases, resulting in recoveries exceeding 100% (101.1 ± 5.3% or 109.5 ± 14.5, respectively). This suggests that the aptasensor was capable of detecting not only S-RBD, but also other components present in the samples that could potentially impede the communication between the sensing layer and the analyte. It appears that the specificity of the aptamer is of significant importance when analyzing complex samples. Prior to testing the aptasensor in real samples, it is essential to conduct a thorough investigation of its performance in buffer solutions. It can be concluded that, for lower concentrations, which are of particular interest in the early detection of infectious diseases, the aptasensor exhibited an optimal response to S-RBD and demonstrated effective control of the matrix effect. The results obtained suggest that the aptasensors exhibit an adequate analytical response toward the S-RBD protein in plasma or saliva samples.

### 3.3. Electrochemical AuE Aptasensor Preparation and S-RBD Detection

Based on the results from the QCM biosensor, an electrochemical aptasensor was constructed using thiol-1C APT as the biorecognition element. This approach aimed to compare the sensitivity, selectivity, detection limits, and preparation time of the electrochemical aptasensor with the QCM biosensor. The one-step modification method was utilized, significantly reducing the preparation time while maintaining the sensitivity for S-RBD detection (see Figure S3).

**Figure 7:**
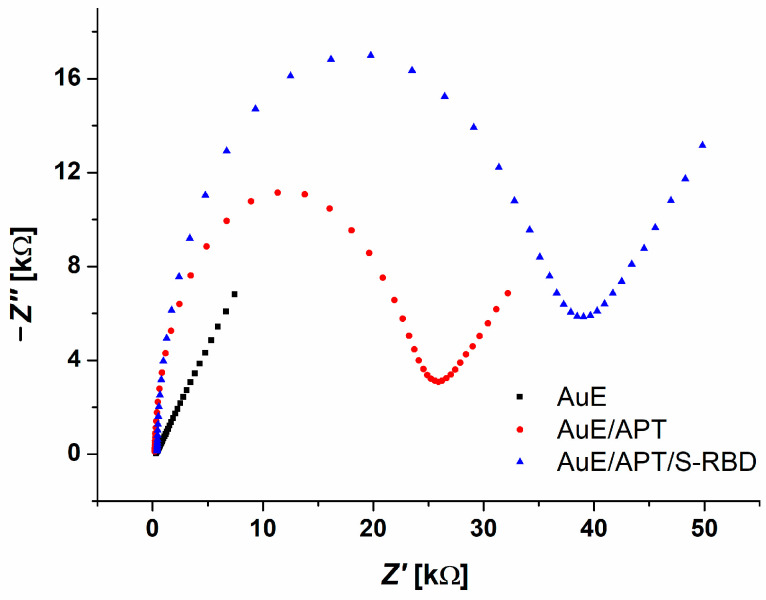
Nyquist diagram of the clean AuE (black), aptasensor (red), and 175 ng/mL S-RBD detection (blue) in redox indicator 1 mM Fe[(CN)_6_]^3−/4−^.

The detection of S-RBD using an electrochemical aptasensor was performed as follows. The prepared aptasensor was incubated for 5 min in a solution of PBS to stabilize the DNA strands. Subsequently, it was connected to a three-electrode system, which was immersed in an electrochemical cell containing an aliquot amount of S-RBD and 1 mM redox indicator Fe[(CN)_6_]^3−/4−^. Analysis was performed using electrochemical impedance spectroscopy (EIS) in the range 10 kHz–0.1 Hz, with an amplitude of 10 mV and a potential of +0.25 V, thereby obtaining information on the binding of the analyte to the aptamer. EIS allows for sensitive label-free detection based on changes in electrical properties at the electrode–solution interface upon target binding [22]. The detection of S-RBD was evidenced by a significant increase in resistance on the electrode surface after protein binding, even at low concentrations (Figure 7, blue curve).

To determine the linear response range of the aptasensor, it was immersed in S-RBD solutions with concentrations ranging from 175 to 5250 ng/mL. Resistance values were determined using EIS, and a plot of S-RBD concentration versus “Δ ratio” (calculated as a share between charge transfer resistance values after and before the aptasensor incubation in S-RBD, related to clean AuE) was constructed. With increasing S-RBD concentration, the EIS records (Nyquist diagrams) showed a proportional increase in electron transfer resistance on the electrode surface up to an S-RBD concentration of 1225 ng/mL. At 1750 ng/mL, the linear relationship was lost, indicating saturation of the protein on the aptasensor surface (see Figure S4). Saturation likely caused some S-RBD to be pulled from the electrode surface, reducing resistance as electron transfer was again possible in those areas.

The method’s LOD was determined to be 132 ng/mL, and the LOQ was 441 ng/mL, based on the linear portion of the calibration curve (Figure S4, Inset).

### 3.4. Selectivity Tests

To confirm the specificity of the aptasensor, selectivity tests were conducted using two competitive proteins, histone H1 (H1) and bovine serum albumin (BSA), both at a concentration of 175 ng/mL. H1 has a comparable molecular weight to S-RBD (33 kDa vs. 35 kDa), while BSA has a larger mass (66.5 kDa). Measurements were performed similarly to the S-RBD detection. Figure S5 shows that the aptasensor exhibited a very low affinity towards BSA, indicating negligible non-specific interactions compared to S-RBD (green vs. red bar/curve). The interaction with H1 caused a significant decrease in resistance, likely due to the aptamers being pulled away from the electrode surface by the large molecular weight of H1 (orange vs. red bar/curve). The “Δ ratio” parameter followed the EIS curve trends, confirming the changes in resistance during aptasensor–protein interactions. No nonspecific interactions were observed, demonstrating the high selectivity of the prepared electrochemical aptasensor.

In Table 2, the comparison between the performance of our biosensors and other selected sensors is shown.

The performance of our biosensors was evaluated and compared with various types of sensors reported in the literature (Table 2). The aptamer-based QCM sensor demonstrated an exceptional LOD of 70 fg/mL, which is among the lowest in the table, indicating superior sensitivity. This LOD is markedly lower than that of the electrochemical SPCE aptasensor (66 pg/mL) [36] and the CRISPR/Cas12a-derived electrochemical aptasensor (16.5 pg/mL) [39], and it is comparably sensitive to the nanoparticle surface energy transfer assay (130 fg/mL) [38]. Moreover, the aptamer-based QCM sensor exhibited a broad linear range, spanning from 1 pg/mL to 0.1 µg/mL, thereby offering versatility in detecting various concentrations of the target analyte. In contrast, the aptamer-based electrochemical AuE biosensor used in our work exhibited a higher LOD of 132 ng/mL, indicating a lower sensitivity relative to our QCM biosensor and the other biosensors listed. The linear range of the aptamer-based electrochemical AuE extends from 175 ng/mL to 5 µg/mL, which is more limited at lower S-RBD protein concentrations in comparison to the CRISPR/Cas12a-derived electrochemical aptasensor (50 pg/mL to 100 ng/mL) and the electrochemical SPCE aptasensor (1 pM to 100 nM) [42]. Although the aptamer-based electrochemical AuE exhibits a relatively higher LOD, it may be a suitable option for applications where the detection of extremely low concentrations is not a critical requirement. In conclusion, the aptamer-based QCM sensor developed in this study exhibits excellent sensitivity and a broad linear range, making it a competitive alternative to some of the most advanced sensors currently available. Conversely, the aptamer-based electrochemical AuE, though less sensitive, may still be applicable in specific contexts where its detection limits are sufficient.

## 4. Conclusions

The paper provides a comparative analysis of two different biosensor technologies—Quartz Crystal Microbalance (QCM) and Electrochemical Impedance Spectroscopy (EIS). This dual approach not only demonstrates the versatility of the aptamer-based detection system, but also highlights the strengths and potential applications of each method.

Both QCM and electrochemical aptasensors exhibit high sensitivity and selectivity for the SARS-CoV-2 S-RBD protein. The QCM aptasensor demonstrated detection at extremely low concentrations (0.07 pg/mL), while the electrochemical aptasensor had a low limit of detection (LOD) of 132 ng/mL. The specific interactions were carefully verified with controls and non-specific proteins, ensuring the reliability and specificity of the sensors.

The electrochemical aptasensor preparation was significantly optimized to a one-step modification process, which drastically reduced the preparation time to approximately 2 h. This is a substantial improvement over traditional methods that can take several hours to over a day. The development of a simplified, rapid assembly process for the electrochemical aptasensor streamlines the sensor fabrication while maintaining high performance. This approach reduces the complexity and time required for sensor preparation. The current validation of the sensors was limited to a controlled PBS environment. This was due to initial steps focusing on optimizing sensor preparation and demonstrating sensitivity and selectivity under well-defined conditions. However, testing in biological fluids is essential for further validation of its potential clinical utility. The same goes for specificity testing against a broader range of clinically relevant antigens, such as proteins from influenza, rhinoviruses, and adenoviruses, which would provide a more comprehensive evaluation of the aptasensor’s specificity. This is particularly important for ensuring the sensor’s practical utility in differentiating SARS-CoV-2 from other common respiratory pathogens.

On the other hand, the QCM aptasensor was tested and validated in biological fluids such as diluted human plasma and saliva. This real-world application underscores the practical utility of the sensor in complex biological environments, an important step towards real-world diagnostic use.

These contributions demonstrate the paper’s advancements in the field of aptamer-based biosensors, providing insights into efficient sensor preparation, high-performance detection, and practical application in real-world scenarios.

## Figures and Tables

**Table 1:** Aptasensor performance in saliva and plasma, respectively.

*c*_S-RBD_ [pg/mL]	Δ*f*_*PBS*_ [Hz]	Δ*f*_*saliva*_ [Hz]	Δ*f*_*plasma*_ [Hz]	Recovery_saliva_ [%]	Recovery_plasma_ [%]
10^2^	30.2 ± 6.4	29.3 ± 2.5	29.3 ± 1.5	93.6 ± 3.6	97.4 ± 5.1
10^5^	65.2 ± 5.9	71.3 ± 9.5	66.0 ± 3.6	109.5 ± 14.5	101.1 ± 5.3

**Table 2:** Comparison of the performance of our biosensors with those in other works and types of biosensors.

Type of Sensor	LOD	Linear Range	Reference
Electrochemical SPCE aptasensor	66 pg/mL	10 pM–25 nM	[36]
Optical sandwich aptamer assay	21 ng/mL	------------	[37]
Nanoparticle surface energy transfer assay	130 fg/mL	10–500 virus mL^−1^	[38]
CRISPR/Cas12a-derived electrochemical aptasensor	16.5 pg/mL	50 pg/mL–100 ng/mL	[39]
Al_2_O_3_ fabricated mercapto-silane AuNP aptasensor	0.8 ng/mL	2.5–40.0 ng/mL	[40]
Molecular imprinting polymer electrochemical SPCE aptasensor	3.3 PFU mL^−1^	10^1^–10^8^ PFU mL^−1^	[41]
Electrochemical SPCE aptasensor	1.1 pM	1 pM–100 nM	[42]
Aptamer-based QCM sensor	70 fg/mL	1 pg/mL–0.1 μg/mL	This work
Aptamer-based electrochemical AuE	132 ng/mL	175 ng/mL to 5 μg/mL	This work

## Data Availability

The data presented and analyzed in this study are available on reasonable request from the corresponding author.

## Supplementary Materials

The following supporting information can be downloaded at: https://www.mdpi.com/article/10.3390/bios14090431/s1: Figure S1: A comparison of the sensitivity of aptasensors prepared with different concentrations of thiol–1C APT. The aptasensor’s response to the addition of 1000 pg/mL S-RBD.; Figure S2: Comparison of the change in resonant frequency (Δ*f_s_*) of the aptasensor when using the 1C aptamer after heating (red curve) and without heating (black curve). The aptasensor’s response to the addition of 1000 pg/mL S-RBD.; Figure S3: Comparison of repeatability of cleaning method, 1C aptasensor preparation, and 175 ng/mL S-RBD detection of three consecutive experiments.; Figure S4: Calibration curve of S-RBD electrochemical detection in the concentration range from 5 to 150 nM (175 to 5250 ng/mL) using electrochemical aptasensors (Δ ratio = 0.687 + 0.061c − 6.963 × 10^−4^c^2^ + 2.183 × 10^−6^c^3^, R^2^ = 0.986). The inset represents the linear part of the calibration curve (Δ ratio = 0.832 + 0.004c; R^2^ = 0.990).; Figure S5: Selectivity test of the electrochemical 1C aptasensor (red) towards 175 ng/mL of S-RBD (blue), BSA (green), and H1 (orange) proteins. Nyquist diagrams are presented with corresponding signals, where the black line represents clean AuE.
