# Supplementary material for: Comparative Analysis of QCM and Electrochemical Aptasensors for SARS-CoV-2 Detection
Source: Biosensors (Basel). 2024 Sep 6;14(9):431. doi: 10.3390/bios14090431 (PMC11429642; doi:10.3390/bios14090431)
Supplement: Supplementary file 1 [file biosensors-14-00431-s001.zip › biosensors-3162897-supplementary.pdf]

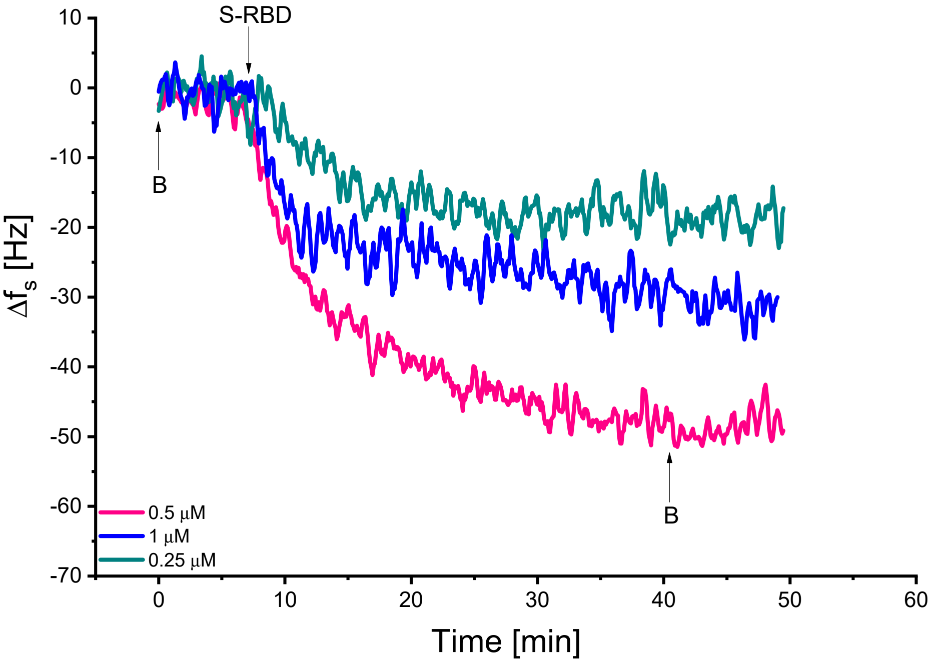

Figure S1. A comparison of the sensitivity of aptasensors prepared with different concentrations of thiol-1C APT. The aptasensor's response to the addition of 1000 pg/mL S-RBD.

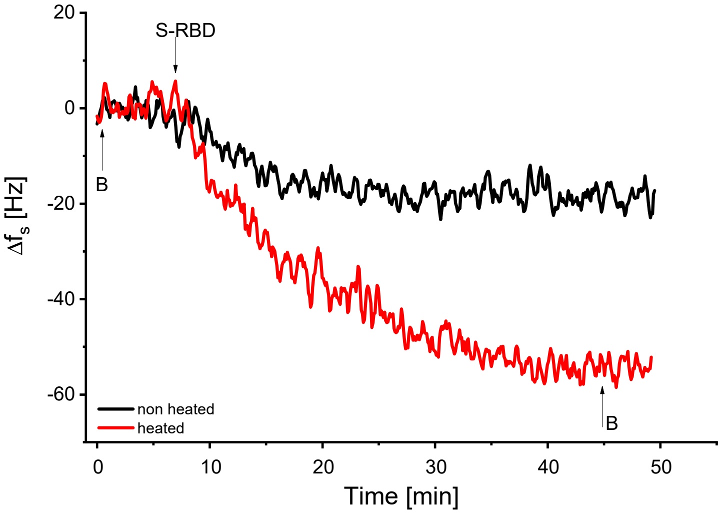

Figure S2. Comparison of the change in resonant frequency (Δf_s) of the aptasensor when using the 1C aptamer after heating (red curve) and without heating (black curve). The aptasensor's response to the addition of 1000 pg/mL S-RBD.

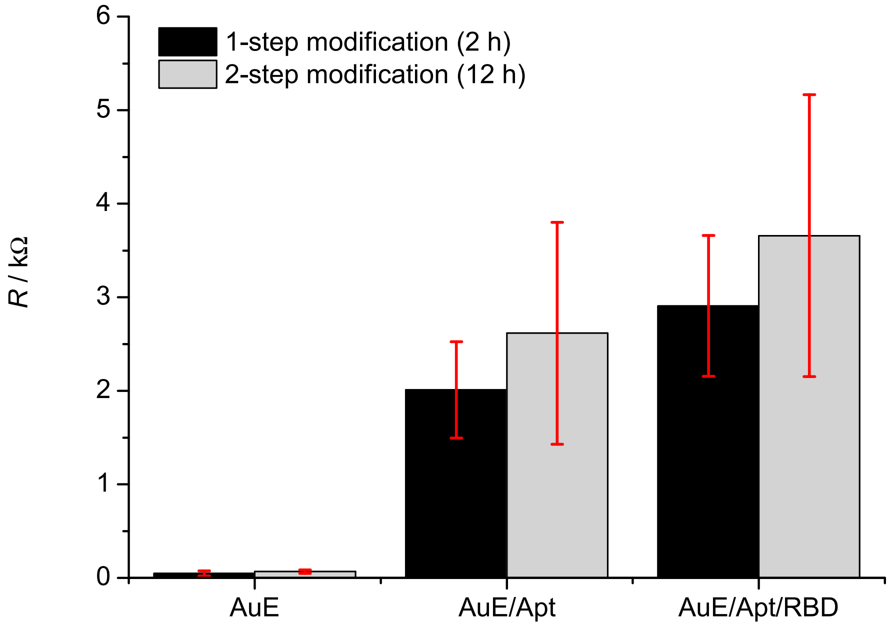

Figure S3. Comparison of repeatability of cleaning method, 1C aptasensor preparation, and 175 ng/mL S-RBD detection of three consecutive experiments.

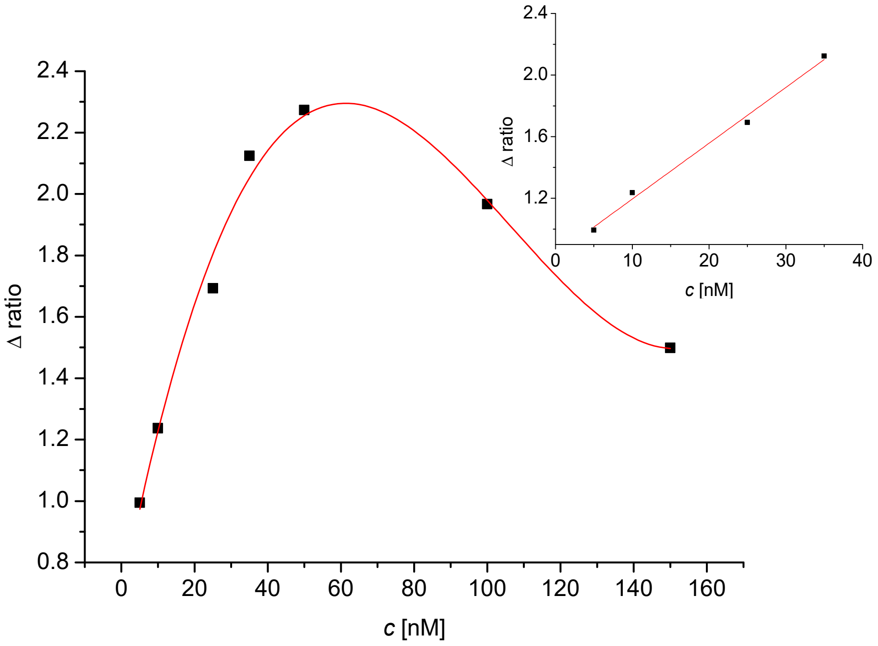

Figure S4. Calibration curve of S-RBD electrochemical detection in the concentration range from 5 to 150 nM (175 to 5,250 ng/mL) using electrochemical aptasensors (Δ *ratio* = 0.687 + 0.061c – 6.963x10$^{-4}c^2$ + 2.183x10$^{-6}c^3$, R^2=0.986). The inset represents the linear part of the calibration curve (Δ *ratio* = 0.832 + 0.004c; R^2=0.990).

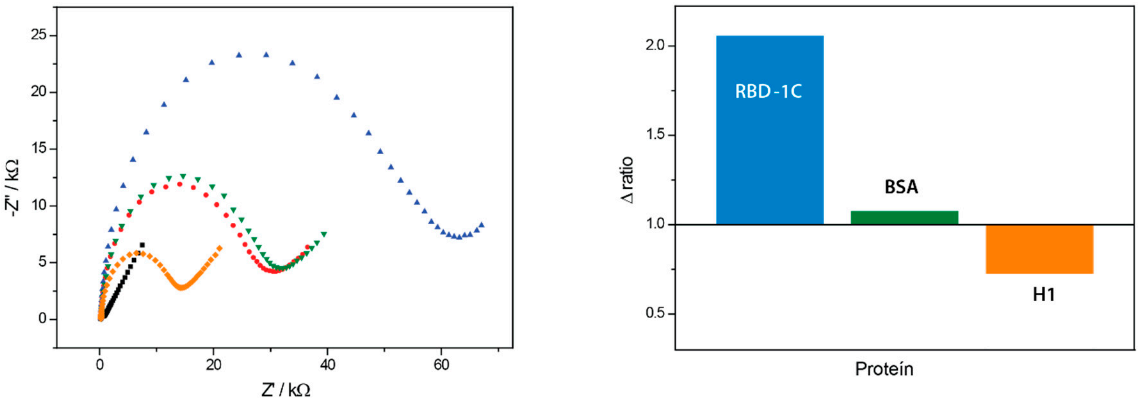

Figure S5: Selectivity test of the electrochemical 1C aptasensor (red) towards 175 ng/mL of S-RBD (blue), BSA (green), and H1 (orange) proteins. Nyquist diagrams are presented with corresponding signals, where the black line represents clean AuE.
